# Polymorphisms on *PAI-1* and *ACE* genes in association with fibrinolytic bleeding after on-pump cardiac surgery

**DOI:** 10.1186/s12871-015-0101-1

**Published:** 2015-09-04

**Authors:** Agnese Ozolina, Eva Strike, Liene Nikitina-Zake, Inta Jaunalksne, Angelika Krumina, Romans Lacis, Lars J. Bjertnaes, Indulis Vanags

**Affiliations:** 1 Department of Cardiac surgery, Pauls Stradins Clinical University Hospital, Pilsonu Street 13, Riga, Latvia; 2 Riga Stradins University, Dzirciema Street 16, Riga, Latvia; 3 Latvian Biomedical Research and Study Center, Ratsupites Street 1, Riga, Latvia; 4 Clinical Immunology Centre, Pauls Stradins Clinical University Hospital, Pilsonu Street 13, Riga, Latvia; 5 Department of Infectology and Dermatology, Riga Stradins University, Dzirciema Street 16, Riga, Latvia; 6 Anesthesia and Critical Care Research Group, Department of Clinical Medicine, Faculty of Health Sciences, University of Tromsø, 9037 Tromsø, Norway

## Abstract

**Background:**

Carriers of plasminogen activator inhibitor -1 (*PAI-1*) -675 genotype 5G/5G may be associated with lower preoperative PAI-1 plasma levels and higher blood loss after heart surgery using cardiopulmonary bypass (CPB). We speculate if polymorphisms of *PAI-1* -844 A/G and angiotensin converting enzyme (*ACE*) intron 16 I/D also might promote fibrinolysis and increase postoperative bleeding.

**Methods:**

We assessed *PAI-1* -844 A/G, and *ACE* intron 16 I/D polymorphisms by polymerase chain reaction technique and direct sequencing of genomic DNA from 83 open heart surgery patients that we have presented earlier. As primary outcome, accumulated chest tube drainage (CTD) at 4 and 24 h were analyzed for association with genetic polymorphisms. As secondary outcome, differences in plasma levels of PAI-1, t-PA/PAI-1 complex and D-dimer were determined for each polymorphism. SPSS® was used for statistical evaluation.

**Results:**

The lowest preoperative PAI-1 plasma levels were associated with *PAI-1* -844 genotype G/G, and higher CTD, as compared with genotype A/A at 4 and 24 h after surgery. Correspondingly, 4 h after the surgery CTD was higher in carriers of *ACE* intron 16 genotype I/I, as compared with genotype D/D. PAI-1 plasma levels and t-PA/PAI-1 complex reached nadir in carriers of *ACE* intron 16 genotype I/I, in whom we also noticed the highest D-dimer levels immediately after surgery. Notably, carriers of *PAI-1* -844 genotype G/G displayed higher D-dimer levels at 24 h after surgery as compared with those of genotype A/G.

**Conclusions:**

Increased postoperative blood loss secondary to enhanced fibrinolysis was associated with carriers of *PAI-1* -844 G/G and *ACE* Intron 16 I/I, suggesting that these genotypes might predict increased postoperative blood loss after cardiac surgery using CPB.

**Electronic supplementary material:**

The online version of this article (doi:10.1186/s12871-015-0101-1) contains supplementary material, which is available to authorized users.

## Background

Heart surgery by means of cardiopulmonary bypass (CPB) can impede hemostasis, thereby increasing postoperative bleeding and the need for blood transfusions [1, 2]. Recently, investigators reported that more than 40 % of the cases of excessive bleedings after this kind of surgery are due to coagulopathy [3]. The balance between bleeding, normal hemostasis and thrombosis is significantly influenced by platelet aggregation, rate of thrombin formation and activation of the fibrinolytic system. Recent evidence suggests that genetic variability might influence the activation of each of these pathways [4, 5].

During CPB, fibrinolytic activity increases 10 - to 100-fold because of augmented generation of plasmin, triggered by a rise in tissue plasminogen activator (t-PA), ultimately resulting in enhanced plasma concentration of fibrin degradation products [6, 7]. Plasminogen activator inhibitor type-1 (PAI-1), the main inhibitor of fibrinolysis, increases a 15-fold only 2 h (h) after cardiac surgery, as a part of the “fibrinolytic shut down” [1, 8]. In a previous study of patients, who underwent cardiac surgery on CPB, we found that lower plasma concentrations of PAI-1 preoperatively were associated with more bleeding, lower levels of t-PA/PAI-1 complex, and higher D-dimer concentrations postoperatively [9].

The PAI-1 gene contains more polymorphisms. The promoter regions 675 4Guanine/5Guanine (4G/5G) and −844 Adenosine/Guanine (A/G) polymorphisms, both affecting the fibrinolytic balance, are two of the most common. Reassessing blood from the latter study [9], we recently confirmed the findings of other investigators that in addition to decreased plasma levels of PAI-1, excessive bleeding after CPB was associated with PAI-1 -675 5G/5G polymorphism [5, 10–14].

Although primarily an endocrine long-term regulator of blood pressure and extracellular volume, the renin-angiotensin-aldosterone system (RAAS) also plays a pivotal role in the regulation of fibrinolysis. Angiotensin converting enzyme (ACE) influences PAI-1 plasma levels by converting angiotensin I to angiotensin II [15, 16]. Recent studies suggest that increased fibrinolysis is mainly related to inhibition of angiotensin II, which acts by reducing the plasma level of PAI-1 rather than increasing that of t-PA [17]. However, plasma concentrations of both PAI-1 and t-PA are characterized by wide inter-individual variations, most likely because of differences in ACE plasma concentrations [18, 19].

Lately, very long half-life PAI-1 seems to represent a promising treatment option for genetically predisposed individuals with low levels of PAI-1 [20]. *PAI-1* gene −844 A/G polymorphism has been associated with both coronary heart disease [21, 22], and venous thrombosis resulting from decreased fibrinolysis [22, 23]. However, to our knowledge, no previous investigation has documented association between *PAI-1* -844 A/G polymorphism and increased blood loss due to increased fibrinolysis after cardiac surgery employing CPB.

Intron 16 Insertion/Deletion (I/D) polymorphism of the *ACE* gene influences concentration of circulating ACE, thereby affecting endogenously generated inhibitor of fibrinolysis. The insertion allele is believed to steer approximately one half of the plasma levels of ACE and PAI-1, and might potentially increase fibrinolytic activity [24]. Several studies have revealed that *ACE* intron 16 I/D polymorphism is accompanied by a wide range of cardiovascular diseases. Genotype D/D is associated both with increased plasma concentration and higher activity of PAI-1 [25–28]. However, the *ACE* Intron 16 I/D polymorphism has been sparsely studied, as a potential cause of postoperative bleeding, and with controversial results [25, 29, 30]. Consequently, by examining the same patient cohort as referred to above [14], our primary end-point was to address the associations between *PAI-1* -844 A/G and *ACE* Intron 16 I/D polymorphisms and fibrinolytic bleeding after cardiac surgery using CPB.

## Methods

Methods has been presented previously in this journal [9] and will only be shortly described. A more extensive version is available, see Additional file 1. The protocol and the informed consent form, including the request to donate genetic material, were approved by the Ethics Committee of Pauls Stradins Clinical University Hospital, Riga, Latvia. All patients provided written informed content.

In short, 90 consecutive adult patients were admitted to the hospital to undergo cardiac surgery with CPB, whereof 7 were excluded at the reoperation because of surgical bleeding. Postoperative bleeding volumes were recorded as chest tube drainage (CTD) in mL at 4 and 24 h after the surgery [9]. Inclusion and exclusion criteria, as well as perioperative management, postoperative bleeding and demographic and laboratory data were the same as published before [9] and genomic DNA was extracted as reported by Ozolina et al. [14]. Here, we only describe the methods used for analysis of PAI-1 -844 A/G and ACE Intron 16 I/D polymorphisms.

### PAI-1 -844 A/G and ACE Intron 16 I/D

Genomic DNA from whole blood of every patient was diluted in 1 ml of water and stored on minus 70 °C until analyzed. The region harboring the *PAI-1* -844 A/G gene polymorphism was amplified using polymerase chain reaction (PCR). The primers had the following sequences: 5′-ATCCCTTTTCCCCTTGTGTC-3′ and 5′-AACCTCCATCAAAACGTGGA-3′. The PCR products were then purified using Sap/Exo I (Thermo Scientific® Fermentas, Lithuania) and sequenced on an ABI Prizm 3130xl genetic analyzer (Applied Biosystems®, Life Technologies, USA).

For determination of *ACE* Intron16 I/D polymorphism, we used the method published by Tomita et al. [31]. Insertion and deletion alleles were identified by using PCR amplification of the respective fragments from Intron 16. Fragment size was determined by agarose gel electrophoresis. The deletion allele was visualized at 191 base pairs (bp), and an insertion allele at 478 bp. For patients with /D genotype additional PCR was performed to verify the result of amplification.

### Statistical analysis

Data were analyzed with SPSS (SPSS® version 20.0, Chicago, IL). Continuous variables were presented as mean ± standard deviation (SD) and categorical variables as percentages (%). The data of the study groups were checked by an appropriate analytic test according to the data distribution. Comparisons between genotype groups were performed with Kruskal-Wallis H test for non-parametric variables, and with ANOVA for parametric variables. Chi-square test was used to analyze categorical data. Statistical significance was defined as *P* < 0.05.

## Results

### Clinical course

Out of totally, 90 consecutive patients scheduled for first time cardiac surgery, 83 patients, 42 men and 41 women, who met inclusion criteria were subjected to further analysis (Table 1). Patients were classified with their *PAI-1-844* A/G and *ACE* Intron 16 I/D characteristics and subdivided into 3 groups, according to the genotype of each polymorphism. The genotype results of *PAI-1* -844 A/G and *ACE* Intron 16 I/D polymorphisms were all in Hardy-Weinberg equilibrium. We noticed no significant differences between CPB priming - and cardioplegia volumes in relation to the different genotypes. We found no associations between *PAI-1 -844* A/G genotype and demographic characteristics, preoperative parameters and surgical variables (Table 1). *ACE* of genotypes I/D and D/D were significantly more represented among males (*P* < 0.05) and in patients undergoing mixed type of surgery (*P* < 0.05).

**Table 1:** Perioperative characteristics of patients scheduled for on-pump cardiac surgery

Characteristics	PAI-1 -844 A/G			ACE Intron 16 I/D		
Genotype	G/G	A/G	A/A	I/I	I/D	D/D
Number of patients, n	22	387	23	22	42	19
Demographic data						
Age, yr	61 ± 11	68 ± 10	67 ± 11	68 ± 10	66 ± 10	63 ± 14
Male sex, n (%)	15 (68)	16 (42)	11 (48)	9 (41)*	23 (55)* **	10 (53)**
BMI, kg/m^2^	28 ± 5	28 ± 8	27 ± 5	27 ± 5	28 ± 5	27 ± 4
EF, (%)	57 ± 8	55 ± 8	56 ± 7	58 ± 6	55 ± 9	55 ± 7
Type of surgery, n (%)						
CABG, n (%)	11 (50)	15 (40)	8 (35)	8 (36)	18 (43)	8 (42)
Valve, n (%)	8 (36)	13 (34)	10 (43)	9 (41)	13 (31)	9 (47)
Mixed, n (%)	3 (14)	10 (26)	5 (22)	5 (23)*	11 (26)* **	2(11)**
Surgical variables						
CPB duration (min)	110 ± 42	106 ± 42	99 ± 37	101 ± 47	105 ± 39	109 ± 37
Aorta oclusion time (min)	69 ± 30	65 ± 25	63 ± 11	64 ± 27	65 ± 27	69 ± 29
Reperfusion time (min)	33 ± 12	33 ± 15	33 ± 16	29 ± 13	36 ± 17	33 ± 9
CPB priming volume, ml	1864 ± 543	1509 ± 516	1329 ± 440	1379 ± 462	1548 ± 556	1544 ± 482
Cardioplegia, ml	1872 ± 587	1673 ± 516	1886 ± 674	1647 ± 523	1833 ± 655	1836 ± 478
Preoperative parameters						
Hemoglobin, g/dL	136 ± 15	139 ± 13	131 ± 18	132 ± 17	137 ± 16	138 ± 12
Platelet count, × 10^9^/L	221 ± 67	215 ± 50	215 ± 63	211 ± 45	218 ± 65	220 ± 58
Prothrombin time, %	84 ± 15	90 ± 14	92 ± 12	94 ± 15	89 ± 13	85 ± 14
Fibrinogen, g/L	4.4 ± 1.3	4.6 ± 1.2	4.7 ± 1.5	4.5 ± 1.2	4.6 ± 1.2	4.7 ± 3.7

Data presented as the mean ± standard error of the mean

*PAI-1* Plasminogen activator inhibitor type-1, *A* Adenosine, *G* Guanine, *ACE* Angiotensin converting enzyme, *I* Insertion, *D* Deletion, *n* Number of patients, *BMI* Body mass index, *EF* Ejection fraction, *CABG* Coronary artery bypass grafting, *CPB* Cardiopulmonary bypass. *P < 0.05 between ACE Intron 16 I/I and I/D genotypes;

**P < 0.05 between ACE Intron 16 D/D andI/D genotypes

### Relationships between *PAI-1* -844 A/G and *ACE* Intron 16 I/D polymorphisms and postoperative bleeding

**Fig. 1:**
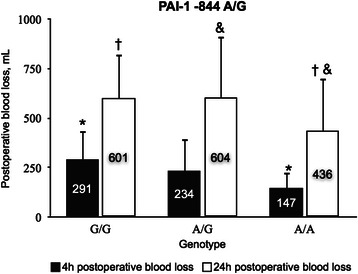
PAI-1-844 A/G polymorphism and blood loss after open heart surgery. Relationship between *PAI-1* -844 A/G polymorphism and accumulated postoperative blood loss after cardiac surgery with cardiopulmonary bypass. Data presented as the mean ± standard error of the mean. PAI-1, Plasminogen activator inhibitor type −1; A, adenosine; G, guanine; mL, milliliters; h, hours. *P < 0.05 4 h blood loss between PAI-1 -844 of genotypes G/G and A/A; † *P* < 0.05 between 24 h blood loss associated with genotypes G/G and A/A of PAI-1 -844; & *P* < 0.05 between 24 h blood loss associated with PAI-1 -844 of genotypes A/G and A/A

Table 2 shows preoperative PAI-1, postoperative t-PA/PAI-1 complex and D-dimer plasma levels in relation to *PAI-1*-844 A/G and *ACE* Intron 16 I/D polymorphisms. Mean PAI-1 plasma concentration was lower in carriers of genotype G/G, as compared to A/A (*P* = 0.004), but no significant difference existed between genotypes A/A and A/G. Mean plasma concentrations of t-PA/PAI-1 complex determined 24 h postoperatively displayed no significant differences between the *PAI-1*-844 genotype groups. In contrast, D-dimer levels differed significantly between carriers of genotypes G/G and A/G (*P* = 0.04) at 24 h (Table 2). As depicted in Fig. 1, carriers of *PAI-1*-844 genotype G/G displayed the greatest blood loss at 4 and 24 h postoperatively, as compared with carriers of genotype A/A (*P* = 0.0001, *P* = 0.03, respectively).

**Table 2:** PAI-1 -844 A/G and ACE Intron 16 I/D gene polymorphisms related to markers of fibrinolysis. Plasma concentrations of PAI-1 preoperatively – and of t-PA/PAI-1 complex and D-dimer postoperatively after on-pump cardiac surgery

Genetic polymorphism	n	PAI-1, ng/mL, preoperatively	t-PA/PAI-1, ng/mL, 24 h postoperatively	D-dimer, ng/mL 0 h	D-dimer, ng/mL 6 h	D-dimer, ng/mL 24 h
PAI-1 -844 A/G						
A/A	23	28 ± 12*	3.8 ± 1.8	251 ± 170	289 ± 210	234 ± 187
A/G	38	24 ± 13	3.6 ± 2.1	312 ± 213	312 ± 200	184 ± 129*
G/G	22	18 ± 12*	3.4 ± 2.4	287 ± 255	255 ± 203	267 ± 168*
*P* value		0.004	NS	NS	NS	0.04
ACE intron 16 I/D						
D/D	19	27 ± 13*	3.6 ± 2	234 ± 161*	244 ± 182	208 ± 173
I/D	42	24 ± 13	4 ± 2.3*	294 ± 238	291 ± 204	247 ± 165
I/I	22	18 ± 11*	2.8 ± 1.7*	376 ± 203*	331 ± 218	274 ± 167
*P* value		0.02	0.02	0.03	NS	NS

Data presented as the mean ± standard error of the mean

*PAI-1* Plasminogen activator inhibitor type-1, *A* Adenosine, *G* Guanine, *ACE* Angiotensin converting enzyme, *I* Insertion, *D* Deletion, *n* Number of patients, *t-PA* Tissue plasminogen activator, *0 h* Immediately after surgery, *6, 24 h* 6 and 24 h postoperatively. *P < 0.05 between genotypes

**Fig. 2:**
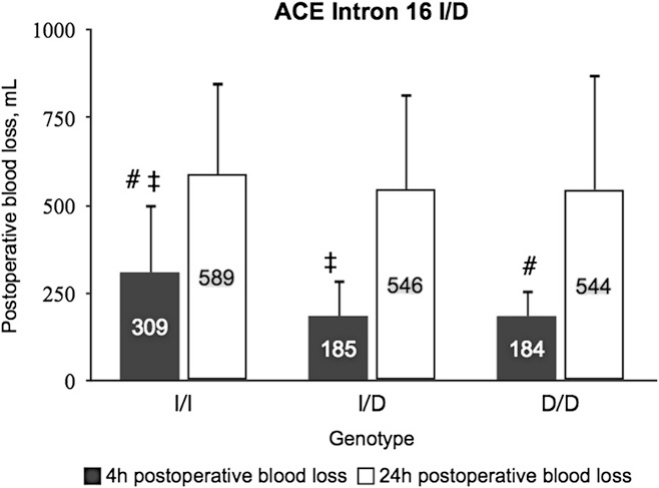
*ACE* Intron 16 I/D polymorphism and blood loss after open heart surgery. Relationship between *ACE* Intron 16 I/D polymorphism and accumulated postoperative blood loss after cardiac surgery with cardiopulmonary bypass. Data presented as the mean ± standard error of the mean. ACE, Angiotensin Converting Enzyme; I, insertion; D, deletion; mL, milliliters; h, hours. # *P* < 0.05 between 4 h blood loss associated with ACE Intron 16 of genotypes I/I and D/D; ‡ *P* < 0.05 between 4 h blood loss associated with ACE Intron 16 of genotypes I/I and I/D

Carriers of *ACE* Intron 16 genotype I/I presented with significantly lower preoperative plasma concentrations of PAI-1, as compared with carriers of genotype D/D (*P* = 0.02), but not as compared with carriers of genotype I/D (Table 2). Concerning t-PA/PAI-1 complex determined 24 h after the surgery (Table 2), we found higher plasma concentrations in carriers of genotypes I/D as compared with I/I (*P* = 0.02). Postoperatively, patients with *ACE* Intron 16 genotype I/I displayed higher plasma concentrations of D-dimer at all three time points. The difference reached significance immediately after the surgery (Table 2) in carriers of genotype I/I in comparison with genotype D/D (*P* = 0.03). Correspondingly, as shown in Fig. 2, carriers of genotype I/I also presented with significantly higher blood loss 4 h after surgery, in comparison with genotypes I/D (*P* = 0.02) and D/D (*P* = 0.04).

## Discussion

This study revealed that blood loss after cardiac surgery with extracorporeal circulation is larger in carriers of *PAI-1* -844 genotype G/G and *ACE* intron 16 genotype I/I as compared with their respective genotypes A/A and D/D. We also noticed the lowest preoperative plasma concentrations of PAI-1 and the highest D-dimer levels 24 h after the surgery in carriers of *PAI-1*-844 genotype G/G. Correspondingly, in carriers of *ACE* intron 16 genotype I/I, D-dimer plasma concentration peaked transiently immediately after the surgery and t-PA/PAI-1 complex reached nadir at 24 h postoperatively.

PAI-1 and t-PA forming t-PA/PAI-1 complex, are supposed to be the main regulators of fibrinolysis in human. According to a recent study, patients with myocardial infarction presented with higher PAI-1 and lower t-PA plasma concentrations as compared with healthy controls [21]. Notably, regression analysis confirmed an independent association between myocardial infarction and genotype -844 A/A in concert with pronounced elevation of PAI-1 [22]. Consistent with the increased anti-fibrinolytic activity associated with myocardial infarction, our findings indicate that genotype A/A rather protects against fibrinolysis, as assessed by a lower accumulated blood loss 24 h postoperatively in carriers of that genotype.

### *PAI-1*-844 A/G polymorphisms

We believe that increased fibrinolysis, as assessed by lower plasma concentrations of PAI-1 and t-PA/PAI-1 complex can explain the augmented blood loss in carriers of *PAI-1-*844 genotype G/G. As compared with heterozygotes and carriers of *PAI-1* -844 A/A, those of genotype G/G presented with significantly larger blood loss and the significantly highest D-dimer plasma concentrations at 24 h. The latter genotype also demonstrated a 36 % reduction in preoperative PAI-1 plasma concentration, as compared with carriers of genotype A/A. This is consistent with observations made by previous investigators in healthy volunteers [32]. Studying *PAI-1*-844 A/G and *PAI-1* -675 (4G/5G) polymorphisms, the latter workers showed that carriers of genotype G-5G had significantly lower plasma concentrations of PAI-1. They also noticed that the plasma level of PAI-1 depends more on body mass index than on PAI-1 promoter variations, a contention we could not confirm in the present study. In carriers of genotype G/G, we also were unable to demonstrate increased fibrinolysis at 0 and 6 h postoperatively, despite the fact that this genotype displayed the significantly highest plasma level of D-dimer 24 h after surgery. We interpret this result as a of lower inhibitory fibrinolytic potential, which is consistent with the “fibrinolytic shut down”, that might occur in parallel with maximum D-dimer levels 24 h after the operation [33].

### *ACE* Intron 16 I/D polymorphisms

Several investigators have focused on a potential association between *ACE* Intron 16 I/D polymorphism and increased postoperative bleeding after cardiac surgery [25, 28–30, 34]. Prior to surgery, we observed 33 % significantly higher preoperative plasma concentrations of PAI-1 in carriers of *ACE* Intron 16 of genotype D/D, as compared with genotype I/I. The latter genotype also displayed significantly lower plasma levels of t-PA/PAI-1 complex and higher levels of D-dimer postoperatively, as compared with genotype I/D. The finding that those with the D-allele displayed the highest plasma levels of PAI-1 agrees with a report evaluating the association between plasma PAI-1 levels and *ACE* Intron 16 I/D polymorphism in healthy volunteers [18]. Despite we observed more blood loss 4 h after the surgery in carriers of genotype I/I, we found no significant differences between the three genotypes 24 h postoperatively. Most likely, the increased blood loss was caused by fibrinolysis. According to previous investigators, plasma concentration of PAI-1 does not rise earlier than 2–3 h after the surgery [1]. The fact that carriers of genotype I/I had the lowest postoperative levels of t-PA/PAI-1 complex (Table 2) strengthens the assumption of an increased fibrinolytic tendency in association with that particular genotype. Other investigators also have reported significant associations between *ACE* 16 I/D polymorphism and postoperative blood loss 12 and 24 h after open heart surgery [28, 29]. In one investigation, the D allele was associated with decreased bleeding consistent with our finding [28]. In contrast, other investigators found larger blood loss 24 h postoperatively in carriers of *ACE* Intron 16 genotype I/I [30].

In carriers of *ACE* Intron 16 genotype D/D undergoing non-cardiac surgery, researchers observed decreased bleeding tendency in association with higher plasma concentrations of ACE [28]. Investigators studying the influence of *ACE* polymorphism on intra – and postoperative bleeding in patients undergoing total hip replacement showed that carriers of D/D and I/D genotypes had the highest total blood losses [25]. In contrast to our findings, these workers suggest that the D allele should be considered as a risk factor of increased bleeding. In their work, patients of genotype I/I displayed higher D-dimer concentrations, suggesting that more efficient activation of coagulation had taken place, consistent with the higher D-dimer levels observed immediately after surgery in the present study. However, the latter investigators did not determine the PAI-1 and t-PA/PAI-1 plasma concentrations that corresponded with the *ACE* 16 I/D polymorphism. Possibly, higher plasma levels of ACE, PAI-1 and t-PA/PAI-1 complex, combined with angiotensin-II-induced increase in vasoconstrictor tone, could explain these findings. Thus, although no general agreement has been reached, we and other investigators support the idea that a greater bleeding tendency might occur in carriers of *ACE* Intron 16 of genotype I/I [29, 30, 34].

### Limitations

Firstly, we admit that the sample size was too low to reach significant difference with, at least 80 % power, and 5 % significance level for analysis of every genetic polymorphism. We compared t-PA/PAI-1 complex plasma concentrations of 22 and 23 patients of genotypes G/G and A/A, respectively, and found that sample sizes of at least 159 patients in each group would be required to reach significant differences between the genotypes (Table 2). We wondered whether simultaneous occurrence of *PAI-1* -844 G/G and *ACE* Intron 16 I/I would give rise to excessive blood loss, or that *PAI-1* -844 A/A and *ACE* Intron 16 D/D would result in less blood loss postoperatively, but sample sizes were too low for such analysis. We also admit as a weakness that we did not include a group of healthy volunteers.

We correlated two gene polymorphisms with the plasma concentrations of individual fibrinolytic factors, but do admit that other confounding factors, like hypothermia, hemodilution, heparin re-bound and platelet damage also might have affected postoperative blood loss after surgery. Some investigators argue that reduction of body temperature lowers endogenous production of PAI-1, thereby giving rise to enhanced fibrinolysis and increased bleeding [35], whereas others refute this idea [36]. We rewarmed the patients to normal body temperature (36.6 °C) before transfer to the recovery rooms. Therefore, it is unlikely that hypothermia reduced the formation of t-PA/PAI-1 complex and increased postoperative bleeding in these patients.

Consumption of coagulation factors and hemodilution (Table 1) also might have contributed to increased blood loss postoperatively [37]. We do not deny, that t-PA/PAI-1 plasma levels occasionally decreased 24 h after the surgery due to the combination of hemodilution and decreased anti-fibrinolytic plasma proteins [6]. However, at 24 h postoperatively, we assume that patients had regained normovolemia because a negative net fluid balance was created upon admission to ICU.

It is hard to distinguish clinically changes in fibrinolysis from coagulation disturbances. We admit as a limitation, that neither euglobulin clot lysis time nor thromboelastography/thromboelastometry (TEG/ROTEM) were performed, although some studies predicate a limited role of the latter tests for detecting fibrinolysis [38, 39]. According to recent studies, TEG/ROTEM can only detect severe fibrinolysis in 5 % of cases as compared to 57 % of the cases of moderate fibrinolysis diagnosed with fibrinolytic markers, such as antiplasmin-plasmin complex [39]. Lower thresholds have been suggested for detecing 30-minute fibrinolysis (LY30) by TEG [38]. Despite the fact that our patients received tranexamic acid during CPB, fibrinolytic markers were analyzed only preoperatively and at 24 h postoperatively. At the latter time point, we assume that 90 % of the anti-fibrinolytic agents were excreted via the urine [40]. Nonetheless, after elimination of other possible causes of bleeding, we found a correlation between our commonly used markers of fibrinolysis and specific genotypes.

## Conclusions

The present study demonstrates that increased postoperative blood loss in patients subjected to cardiac surgery with the use of CPB might be caused by increased fibrinolysis secondary to decreased plasma concentration of PAI-1 due to *PAI-1* -844 G/G or *ACE* Intron 16 I/I polymorphisms. We suggest that screening for genetic polymorphisms might become part of future pre-operative routines in order to prevent risks for postoperative bleeding due to disorders in the coagulation – or the fibrinolytic systems.

## Additional file

Additional file 1: **Supplemental methods.** (PDF 94 kb)

## Abbreviations

*ACE* 16 intron I/D: Angiotensin converting enzyme insertion/deletion gene polymorphism at 16-intron (rs4646994)
ACT: Activated coagulation time
BMI: Body mass index
CABG: Coronary artery bypass grafting
CPB: Cardiopulmonary bypass
CTD: Chest tube drainage
EuroSCORE: European system for cardiac operative risk evaluation
Hb: Hemoglobin
ICU: Intensive care unit
kg: Kilograms
LMWH: Low molecular weight heparin
PAI-1: Plasminogen activator inhibitor type-1
*PAI-1*-675 (4G5G): Plasminogen activator inhibitor −1 gene 4 Guanine/5Guanine polymorphism at position 675 (rs1799768)
*PAI-1*-844 A/G: Plasminogen activator inhibitor type-1 Adenosine/Guanine polymorphism at position 844 (rs2227631)
PCR: Polymerase chain reaction
PLT: Platelets
RAAS: Renin angiotensin aldosteron system
SD: Standard deviation
s: Seconds
TEG/ROTEM: Thromboelastography/thromboelastometry
t-PA: Tissue plasminogen activator
t-PA/PAI-1: Complex of tissue plasminogen activator and plasminogen activator inhibitor type-1
0 h: Time point immediately after surgery
4 h: Time point 4 h after surgery
6 h: Time point 6 h after surgery
24 h: Time point 24 h after surgery
