# Supplementary material for: Polymorphisms on PAI-1 and ACE genes in association with fibrinolytic bleeding after on-pump cardiac surgery
Source: BMC Anesthesiol. 2015 Sep 4;15:122. doi: 10.1186/s12871-015-0101-1 (PMC4560913; doi:10.1186/s12871-015-0101-1)
Supplement: Additional file 1: — Supplemental methods. (PDF 94 kb) [file 12871_2015_101_MOESM1_ESM.pdf]

Supplemental Methods

The Ethics Committee of Pauls Stradins Clinical University Hospital, Riga, Latvia approved the study protocol and the informed consent form (No.151209-4L). We obtained written informed consent from every patient. In addition, on our request, all the patients agreed to donate genetic material to the Genome Database of the Latvian Population.

Population

Ninety consecutive adult patients, who were admitted to the hospital to undergo cardiac surgery by the use of CPB, were considered for a prospective observational study. The demographic and clinical characteristics of the patients are the same as presented in two previous studies where associations between fibrinolytic markers and blood loss were compared between bleeders and non-bleeders [30] as well as the influence of PAI-1 -675 (4G/5G) polymorphism to fibrinolytic activity was investigated [18].

Inclusion criteria: >18 years of age, first-time coronary artery bypass grafting (CABG) and/or valve replacement under CPB, EuroSCORE II [31] < 10%. To identify preoperatively the patients at similar risks of bleeding, those enrolled had baseline coagulation tests within normal ranges, including prothrombin time (PT) 70-120% or international normalized ratio (INR) 0.8-1.2 and fibrinogen plasma concentration 1.5 – 3.5 g/L. Moreover, those included had platelet count (PLT) 150 – 400 x 10^9/L, hemoglobin (Hb) concentration > 135 g/L for men and > 120 g/L for women and no anticoagulant, - anti-aggregating or non-steroidal anti-inflammatory drugs for, at least, five days prior to surgery to exclude medicine-induced platelet dysfunction. The last dose of low-molecular-weight heparin (LMWH) was administered the evening before the surgery. Intraoperative factors such as duration of CPB, volumes of administered priming - and cardioplegia solutions, as well as bleeding tendencies where equally expressed in all polymorphism groups. Immediately after the surgery, kaolin and heparinase- activated thrombo-elastography was performed to exclude residual heparin effects or coagulation abnormalities. Cell-saver blood was discarded for included patients.

Exclusion criteria: emergency - and redo operations, surgical bleeding approved during re-exploration, preoperative hemostatic disorders with a history of hemorrhagic events or

coagulopathy, severe renal and/or hepatic dysfunctions, and patients reluctant to allow their blood to be genetically analyzed.

Perioperative management

Anesthesia was induced with fentanyl (Fentanyl-Kalceks® 0.05mg/ml, A/S Kalceks, Latvia) 0.2-0.3 mg, etomidate (Etomidate®, Sagent Agila, India) 0.1-0.3 mg/kg and cisatracurium (Nimbex® 2mg/ml, GlaxoSmithKline Manufacturig S.p.A, Italy) 0.2 mg/kg intravenously and maintained with inhalation of sevoflurane (Sevoflurane Piramal®,Piramal Healthcare Ltd, United Kingdom) at 0.8-1.2 MAC. All patients received tranexamic acid (Exacyl®, Polfa S.A., Warszawa, Poland) 2 g during the surgery. Before the start of CPB, heparin (Pan-Heparin Sodium®, Panpharma S.A./Rotexmedica Gmbh, Germany) 300 - 400 units/kg followed by 5.000 - 10.000 units was administered to maintain activated coagulation time (ACT) above 480 seconds. During CPB (Admiral®, Eurosets TM, Italy), anesthesia was maintained with fentanyl 0.05-0.1 μg/kg/min, propofol 3-5 mg/kg/h and cisatracurium 0.1 mg/kg/h. The extracorporeal circuit was primed with saline solution. Cardioplegic solution was given for myocardial protection (St. Thomas 4:1 cardioplegia; AlleMan, Germany) and dosed depending on the physical constitution of the patient and the duration of CPB. Patients were cooled to a bladder temperature of 34-35 ^{o}C. Packed red blood cells were administered to the extracorporeal circuit if the baseline hematocrit was below 35% [39]. Weaning off CPB was performed after the patient was rewarmed to a bladder temperature above 36 ^{o}C. After separation from CPB, protamine (Protamin Meda®, Meda Pharma, Austria) 1 mg per 100 units of heparin was administered. ACT was determined 4 and 8 h after the surgery to unmask heparin re-bound effects, and if necessary, additional protamine was given. Cryoprecipitate was transfused at a dose of 1 unit per 10 kg immediately after weaning off CPB if the baseline fibrinogen level was less than 2.5 g/L [1].

Demographic and laboratory data

The following variables were registered: age, gender, body mass index (BMI), ejection fraction (EF), type of surgery, time on CPB, aorta clamp - and reperfusion times (min), hemoglobin concentration (Hb, g/L) platelet count (PLT, x 10^9/L) and fibrinogen (g/L). Fibrinogen plasma concentration was determined according to Clauss [32]. Prothrombin (PT) was analyzed with a complex assay (Lyophilized Dade® and Innovin®, Siemens Healthcare Diagnostics, USA). All the

coagulation tests were determined using Sysmex® CA-1500 (Siemens Healthcare Diagnostics, Germany). Hb and PLT were analyzed by means of a Beckman Coulter LH 750 Hematology Analyzer.

Two fibrinolysis markers were assessed: preoperative PAI-1 and t-PA/PAI-1 complex 24 h postoperatively. PAI-1 (normal range 1-25 ng/mL) and t-PA/PAI-1 complex (normally < 5 ng/mL) were determined by using enzyme-linked immunosorbent assay (ZYMUTEST, HYPHEN BioMed, France). Cross-linked fibrin degradation products (normally < 300 ng/mL) were quantified with the immunoturbidimetric test (D-dimer PLUS, Dade Behring, Marburg, Germany) at 3 time points postoperatively: immediately after surgery (0 h), and at 6 h and 24 h postoperatively, respectively.

Genomic DNA was extracted from whole blood by using a standard fenol-chlorophorm extraction technique. DNA was then diluted in 1 ml of water and stored for future analysis. The region harboring the *PAI-1* -844 A/G gene polymorphism was amplified using polymerase chain reaction (PCR). The primers had the following sequences: 5'-ATCCCTTTTCCCCTTGTGTC-3' and 5'-AACCTCCATCAAAACGTGGA-3'. The PCR products were then purified using Sap/Exo I (Thermo Scientific® Fermentas, Lithuania) and sequenced on an ABI Prizm 3130xl genetic analyzer (Applied Biosystems®, Life Technologies, USA).

For determination of *ACE* Intron16 I/D polymorphism the method described by Tomita et al [33] was used. Insertion and deletion alleles were identified by using PCR amplification of the respective fragments from Intron 16. Fragment size was determined by agarose gel electrophoresis. The deletion allele was visualized at 191 base pairs (bp), and an insertion allele at 478 bp. For patients with /D genotype additional PCR was performed to verify the result of amplification due to the possible preferential amplification of deletion allele.

Postoperative bleeding

The postoperative bleeding volumes were recorded as chest tube drainage (CTD) in mL 4 h and 24 h after the surgery. The CTD system was connected to an aspiration pump providing a suction of 15 cm H_2O. Indication for reoperation because of suspected surgical bleeding was based on a rate of blood loss > 100 mL/h or > 2 mL/kg in the first 4 h after surgery in combination with clinical

and hemodynamic changes. A surgical bleeding was ultimately diagnosed at the time of re-exploration. Patients with diagnosed surgical bleeding during reoperation were excluded from further study.

Statistical analysis

Data were analyzed with SPSS (SPSS® version 20.0, Chicago, IL). Continuous variables were presented as mean ± standard deviation (SD) and categorical variables as percentages (%). The data of the study groups were checked by an appropriate analytic test according to the data distribution. Comparisons between genotype groups were performed with Kruskal-Wallis H test for non-parametric variables, and with ANOVA for parametric variables. Chi-square test was used to analyze categorical data. Statistical significance was defined as $P < 0.05$.
